# Taxonomic Revision of the Genus *Dianthus* (Caryophyllaceae) in Tunisia (North Africa)

**DOI:** 10.3390/plants14172720

**Published:** 2025-09-01

**Authors:** Gianniantonio Domina, Ridha El Mokni, Fortunato Cirlincione, Giulio Barone

**Affiliations:** 1 Department of Agricultural, Food and Forest Sciences, University of Palermo, Viale delle Scienze, Bldg. 4, 90128 Palermo, Italy; gianniantonio.domina@unipa.it (G.D.); giulio.barone01@unipa.it (G.B.); 2 Laboratory of Botany, Cryptogamy and Plant Biology, Department of Pharmaceutical Sciences “A”, Faculty of Pharmacy of Monastir, Monastir University, Avenue Avicenna, Monastir 5000, Tunisia; 3 Laboratory of Forest Ecology, National Research Institute of Rural Engineering, Water and Forests, IRESA, Carthage University, Ariana 2080, Tunisia; 4 Department of Soil, Plant and Food Sciences, University of Bari Aldo Moro, Via G. Amendola, 165/A, 70126 Bari, Italy

**Keywords:** taxonomy, nomenclature, herbaria, Mediterranean flora

## Abstract

This study presents a taxonomic revision of the genus *Dianthus* (Caryophyllaceae) in Tunisia, addressing the nomenclatural and morphological complexities of this taxon. Through extensive herbarium research and field investigations, the authors confirm the presence of seven species in Tunisia, including *D. cintranus* (subsp. *byzacenus*), *D. crinitus*, *D. illyricus* (subsp. *angustifolius*), *D. nudiflorus*, *D. rupicola* (subsp. *hermaeensis*), *D. serrulatus*, and *D. virgineus*. Lectotypes are designated for 11 previously unresolved names, clarifying taxonomic ambiguities. The study challenges existing classifications, particularly for *D. serrulatus*, where subspecies distinctions are deemed untenable due to overlapping distribution and morphological traits. For *D. virgineus*, the rank of variety is proposed for the three subspecies present in the country, reflecting the observed morphological diversity. Additionally, six taxa previously reported in Tunisia, such as *D. balbisii* and *D. ferrugineus*, are excluded due to a lack of evidence confirming their presence. The research clarifies the nomenclature of taxa previously reported in Tunisia and reports the distribution of species actually found. An identification key is also given. This revision provides a foundational update for future studies on *Dianthus* in North Africa.

## 1. Introduction

The genus *Dianthus* L. (Caryophyllaceae) ranges mainly in the temperate regions of the Old World. It includes between 300 and 384 species, and many of them are narrow endemics [1,2]. *Dianthus* has undergone taxonomic investigations since the 18th century [3,4,5,6]; however, in many cases the original material used for the description of taxa is still unknown and different nomenclatural types have not yet been designated [7]. In addition, in the last few years, additional new species have been described with little attention given to the names already published [7]. This has created a proliferation of new names, which has further garbled the already complex taxonomic arrangement within this genus [8]. Molecular analyses investigating the broader phylogenetic relationships within this group [2] have shown that the *Dianthus* clade also includes *Velezia* and several *Petrorhagia* taxa.

Integrated taxonomic investigations within the *Dianthus virgineus* L. complex in Europe, with a focus on Italy, are underway [7,9,10]. This approach suggests that the number of genetically recognizable groups in the central Mediterranean is much lower than current taxonomic hypotheses [8,10] and emphasizes the need to embrace even wider geographical territories.

The aim of this study is to revise the taxa of the genus *Dianthus* reported in Tunisia, in agreement with the taxonomic consensus that is consolidating in the rest of the Mediterranean area.

Overall, 28 specific and infra-specific names are reported in the literature for Tunisia [11,12,13,14,15,16]; these names currently refer to seven taxa [14,15].

Nine of them have an already designated nomenclatural type: *Dianthus caryophyllus* L., *D. ferrugineus* Mill., *D. gasparrinii* Guss., *D. godronianus* Jord., *D. hermaeensis* Coss., *D. longicaulis* Ten., *D. siculus* C. Presl, *D. virgineus* L., and *D. vulturius* Guss. & Ten. In this contribution, the types of the remaining 11 taxa are designated as follows: *D. amoenus* Pomel, *D. balbisii* Ser., *D. boissieri* Willk., *D. broteroi* Boiss. & Reut., *D. byzacenus* Burollet, *D. crinitus* Sm., *D. crinitus* var. *flaviflorus* Emb., *D. mesanidum* Litard. & Maire, *D. serrulatus* Desf., *D. serrulatus* var. *macranthus* Maire, and *D. serrulatus* var. *subsimplex* F.N. Williams ex Maire. An overall taxonomic assessment of the taxa considered, based on the morphology of herbarium data and field checks, is provided.

## 2. Materials and Methods

We checked the scientific literature for the effective place of publication of *Dianthus* names reported in Tunisia. The bibliographic data were retrieved from available digital sources and libraries of European institutions, while the original material was searched in the main European herbaria, ANG, BM, COI, G, K, MPU, NAP, P, PAL, SAF, VTA, and W, with the herbarium acronyms to follow [17]. A preliminary online screening was made possible thanks to digital images of herbarium specimens provided by GBIF (https://www.gbif.org, accessed on 1 June 2025), Jstor (http://plants.jstor.org, accessed on 1 June 2025), and ReColNat (https://www.recolnat.org/fr/, accessed on 1 June 2025).

A major part of Maire’s relevant original material is kept in the herbarium of the Université Montpellier 2 (MPU); duplicates are housed in the Muséum National d’Histoire Naturelle in Paris (P) [18].

**Figure 1:**
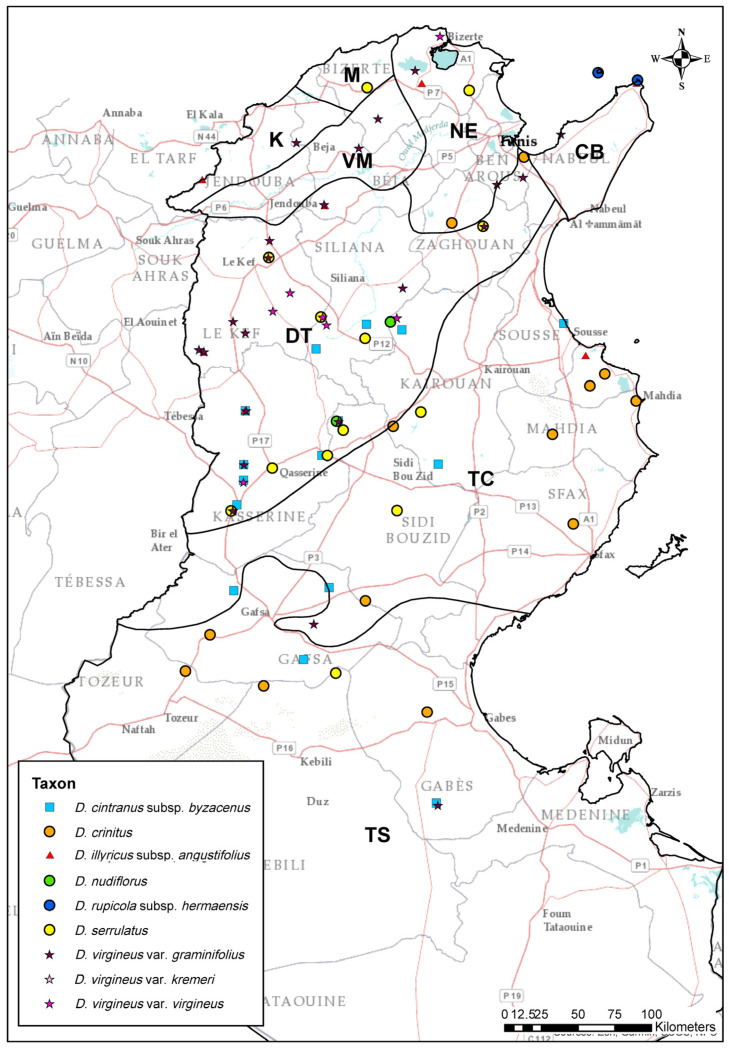
Distribution map of *Dianthus* taxa present in Tunisia according to the studied specimens. Biogeographical areas of the country follow [21].

The articles of the International Code of Nomenclature for Algae, Fungi, and Plants (hereafter ICN) follow [19]. Our taxonomic delimitations, based on morphological comparisons should be considered accepted until more in-depth integrated taxonomic investigations are completed. In this group, the key characters that most effectively distinguish between species [20] include leaf length and width, number of flowers per scape, and shape and length of epicalyx scales. These traits were used to assess the morphological characteristics of the selected types. Additionally, petal characters were suggested as useful discriminative features [20]; however, these are best observed in fresh specimens rather than herbarium samples. A key to the identification of Tunisian taxa of *Dianthus* was developed. New herbarium specimens were deposited in PAL, SAF, in the herbarium of the University of Carthage, and in the personal herbarium of Ridha El Mokni (Herb. R. El Mokni) housed at the Faculty of Pharmacy of Monastir within Monastir University (the last two are not yet listed in the Index Herbariorum). For each taxon, the following information was provided: the accepted name (in bold), homotypic synonyms, nomenclatural type, locality indication (when the type is designated here), nomenclatural notes, heterotypic synonyms, taxonomic and general distribution notes, distribution in Tunisia with details of biogeographical areas—Cap Bon (CB), Dorsale Tunisienne (DT), Kroumirie (K), Mogods (M), Nord-Est (NE), Tunisie Centrale (TC), Tunisie du Sud (TS) and Vallée de la Medjerda (VM) (according to [21])—and specimina visa, arranged by date of collection. A distribution map of the taxa present in Tunisia according to the studied specimens is provided in Figure 1.

## 3. Results

### 3.1. Taxa Occurring in Tunisia

**Figure 2:**
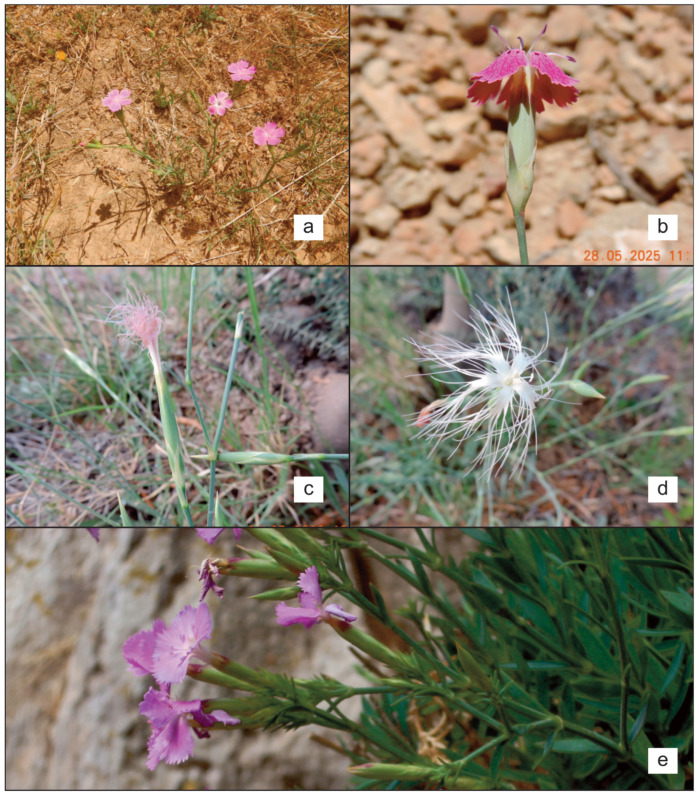
Field photos of *Dianthus* taxa present in Tunisia: (**a**,**b**) *D. cintranus* subsp. *byzacenus* in Dj. Bireno; (**c**,**d**) *D. crinitus* in Hajeb El Ayoun; (**e**) *D. rupicola* subsp. *hermaeeinsis* in Zembra (Photos by R. El Mokni).

***Dianthus cintranus*** subsp. ***byzacenus*** (Burollet) Greuter & Burdet, Willdenowia 12: 186. 1982 (Figure 2a,b).

≡ *D. byzacenus* Burollet, Sahel Sousse 35. 1927.

≡ *D. gaditanus* subsp. *byzacenus* (Burollet) Maire, Fl. Afr. Nord 10: 307. 1963.

**Ind. Loc.**: Nord de Hammam-Sousa, Hammam-Sousa, Sidi-el-Hani, Zeram-el-Din [Zeramdine], Ksar-el-Sef [Ksour Essef], Oulèd Meter, Kessera, Kef Rezaï, Oued Marguellil, Aïn Meghila, Foum El-Guelta, entre Foum El Guelta et Sbeitla, Dj. Chambi, Dj. Khechem-el-Kelb, Dj. Hattig, Dj. Sened, El-Aïeïcha, Oued Tebaga, Dj. Berd, Dj. Mezemzem, Bir El Ahmar.

**Type** (**lectotype designated here**): TUNISIA. Dj. Khechem-el-Kelb, in pinèdes, 23 June 1884, *A. Letourneux et J. F. Robert s.n.* (P04966180 [digital photo!]).

**Nomenclatural notes**: Many specimens cited in [22] (sub. *D. campestris*) and in the protologue (syntypes) are housed in P: P04966180, P04966182, P04966183, P04966184, P04966185, P04966186, P04966187, P04966188, P04966190, etc. Here, we designated the most complete and well-conserved one as the lectotype of the name. The lectotype designated here matches the protologue and corresponds to the current application of the name, referring to a taxon of *D.* section *Tetralepides leiopetala* sensu [5,6].

– *D. campestris* sensu Barratte in Bonnet & Barratte, Expl. Sci. Tunisie, Cat. Pl. 48. 1896 non M.Bieb. in Fl. Taur.-Caucas. 1: 326. 1808.

**Taxonomy and distribution**: *Dianthus cintranus* differs from *D. campestris* by having a cylindric epicalyx, not enlarged at the base, with more deeply incised teeth (1/3 of the epicalyx long vs. 1/5). *Dianthus cintranus* subsp. *byzacenus* differs from *D. cintranus* subsp. *cintranus,* endemic to Portugal, by having shorter leaves, 2–3 vs. 4–6 cm long, and erect, rigid, and non-flexible scapes. It is endemic to Tunisia. Currently, nine subspecies of *D. cintranus* are recognized [14,23]: two are endemic to Portugal (*D. cintranus* subsp. *cintrnaus* and *D. cintranus* subsp. *barbatus* R.Fern. & Franco), six to Morocco and Algeria (*D. cintranus* subsp. *atrosanguineus* (Emb. & Maire) Greuter & Burdet, *D. cintranus* subsp. *jahandiezii* (Maire) Greuter & Burdet, *D. cintranus* subsp. *maroccanus* (F.N.Williams) Greuter & Burdet, *D. cintranus* subsp. *mauritanicus* (Pomel) Greuter & Burdet, *D. cintranus* subsp. *mentagensis* (Maire) Greuter & Burdet, *D. cintranus* subsp. *occidentalis* (Quézel) Mathez) and one to Tunisia (*D. cintranus* subsp. *byzacenus*). These taxa are distinguished by flower size, petal color, and the shape of the epicalyx scales. A focused study of this complex, combining original material and field observations, is needed to verify this taxonomic hypothesis.

**Distr. in Tunisia** (Figure 1): TC: Kairouan (Oueslatia), Sousse (Hammam-Sousse); DT: Kasserine (Dj. Bireno, Dj. Chambi, Dj. Khechem-el-Kelb, Fériana, Sbeitla, Thélepte), Kasserine & Sidi Bouzid (Dj. Mghila), Siliana (Kef Errai, Kessera); TS: Gabès (Matmatas), Gafsa (Dj. Berda, Dj. Attig/Hattig, Dj. Sened), Tataouine (Bir El-Ahmar).

**Ecology and phenology in Tunisian populations**: Rocky soils, fields and roadsides in coastal and hilly areas. Flowering from late April to July.

**Specimina Visa**: Kef Er Rezai, 7 June 1883, *E. Cosson et al. s.n.* (P04966184, P04966185); Nord d’Hammam Sousa, 7 June 1883, *E. Cosson s.n.* (VTA045271); Nord de Hammam-Sousa, 4 June 1883, *E. Cosson et al. s.n.* (P04966183); Djeb. Sened, 1 May 1884, *N. Doumet-Adanson & E. Bonnet s.n.* (P04966188); Bir El-Ahmar, 4 May 1884, *A. Letourneux s.n.* (P04966190); Djeb. Hattig, 17 May 1884, *N. Doumet-Adanson & E. Bonnet s.n.* (P04966187); 19 May 1887, *A. Letourneaux s.n.* (P04966186); Djeb. Bereda, 29 May 1884, *N. Doumet-Adanson & E. Bonnet s.n.* (P04966191); Dj. Khechem-el-Kelb, 23 June 1884, *A. Letourneaux et J. F. Robert; s.n.* (P04966180); Prope Haouaria, 1884, *A. Letourneux s.n.* (P04966189); Inter Sbeitla et Foum El-Guelta, Kef Er Rezai, 24 May 1887, *A. Letourneaux s.n.* (P04966182); Tunesien: 6 km E Thelepte; 770 m, 15 May 1982, *H. Malicky s.n.*, (W0080241); Matmatas, 1 December 2014, *R. El Mokni 3* (Herb. R. El Mokni, SAF); Dj. Mghilla, 25 April 2017, *R. El Mokni 8* (Herb. R. El Mokni, SAF); Oueslatia (Kairouan), 27 April 2017, *R. El Mokni 7* (Herb. R. El Mokni, SAF); Near Djebel Chambi, 35°9′47”N 8°39′47”E, 925 m a.s.l., 27 May 2022, *G. Domina, R. El Mokni, G. Barone s.n.* (Herb. R. El Mokni, SAF), Dj. Bireno, 28 May 2025, *R. El Mokni s.n.* (Herb. R. El Mokni).

***Dianthus crinitus*** Sm., Trans. Linn. Soc. London 2: 300. 1794 (Figure 2c,d).

**Ind. Loc.**: Armenia.

**Type** (**lectotype designated here**): Ex Oriente. Herb. Tourn (LINN-HS 813.32 [digital photo!]).

**Nomenclature notes:** In the protologue of *Dianthus crinitus* is reported “variat flore albo Tourn.”. We found a specimen in the Smith herbarium with white or light pink petals (LINN-HS 813.32) from “Oriente.” This specimen, designated here as the lectotype of the name, includes a portion of plant with three flowering stems and basal leaves. It matches the protologue and corresponds to the current application of the name, which refers to a species occurring in Central Asia and neighboring regions.

= *D. amoenus* Pomel, Bull. Soc. Sci. Phys. Algérie 11: 210. 1874.

*= D. crinitus* var. *amoenus* (Pomel) Maire, Fl. Afrique N. 10: 300. 1963.

Ind. Loc.: [Algeria] Rochers schisteux: Garrouban.

Type (lectotype designated here): ALGERIA. Rochers schisteux: Garrouban, 15 July 1855, *Pomel s.n.* (MPU005112 [digital photo!]; iso: MPU005113 [digital photo!]).

Nomenclatural notes: In MPU we found two specimens (MPU005112 and MPU005113) of *Dianthus amoenus*, both collected by Pomel on 15 July 1855 from Ghar-Rouban rochers, and both corresponding to the description in the protologue. Here, we selected the specimen MPU005112 as the lectotype of the name because it was more complete.

= *D. mesanidum* Litard. & Maire, Mém. Soc. Sci. Nat. Maroc 4: 7. 1924.

= *D. serratulus* subsp. *macranthus* var. *mesanidum* (Litard. & Maire) Maire, Fl. Afr. Nord 10: 303 (1963).

Ind. Loc.: “in rupibus calcareis in convalle Reraya Atlantis Majoris: inter Asni et Tinitine, ad alt. 1350 m”.

Type (lectotype designated here): MOROCCO. Universitè d’Alger, Herbier de l’Afrique du Nord, *Dianthus mesanidum* n. sp., M. Grand Atlas Reraya: rochers, calcaire entre Asni et Tinitine, 1350 m, 20 July 1922, *R. Maire s.n.* (MPU000272 [digital photo!]).

Nomenclatural notes: We found in MPU one herbarium specimen collected by Maire in Reraya, in the Algerian Atlas, in 1922. It is complete in all of its parts and coincides with the description and with the current use of the name.

= *D. crinitus* var. *flaviflorus* Emb., Bull. Soc. Sci. Nat. Maroc. 15: 196. 1935.

Ind. Loc.: Haute Moulouya: steppe d’Halfa près Tamalout, 1700 m, sol argilo-calcaire.

Type (lectotype designated here): MOROCCO. Haute Moulouya: steppe d’Halfa le long de la piste de Midelt à Tounfit, 1700 m, 2 July 1934, *L. Emberger s.n.* (MPU006112 [digital photo!]).

Nomenclatural notes: We found two herbarium sheets in MPU with the original material of the name, MPU006112, includes seven flowerings stems and has a handwritten label by Emberger, while MPU006113 includes seven flowering stems only and probably belongs to the same collection. Here, we prudentially designated MPU006112 as the lectotype of the name because it is the most complete one. This specimen matches the protologue and corresponds to the current application of the name, which is a synonym of *D. crinitus* s.s.

– *D. crinitus* var. *australis* Maire, Fl. Afrique N. 10: 299. 1963 [nom. illeg].

Nomenclatural notes: This name was nomenclaturally superfluous when published because it included the type of *D. crinitus*.

**Taxonomy and distribution**: *Dianthus crinitus* belongs to *D.* sect. *Fimbriatum* subsect. *Gonaxostolon* [23]. It differs from *D. serrulatus* by having mucronate epicalyx scales (not acuminate) and petals with longer fimbria (longer than 4 mm). It is an Irano-Turanian species occurring in North Africa, the Middle East, and Central Asia [24].

**Distr. in Tunisia**: Widespread: TC: Kairouan (Hajeb El Ayoun), Sfax (Bir Arrach), Sidi Bouzid, Mahdia (El-Jem), Monastir (Touza, Zeramdine); DT: Ben Arous (Dj. Bou Kournine), Zaghouan (El Fahs); TS: Gabès (Bir Béni Zid), Gafsa (Aïn Segoufta, Metlaoui), Tozeur (Gouifla), Kebili (Dj. Torrich).

**Ecology and phenology in Tunisian populations**: Sandy soils, fields and roadsides in coastal and hilly areas. Flowering from April to July.

**Specimina visa**: Bir Arrach, 19 April 1884, *N. Doumet-Adanson et Bonnet s.n.* (P04966860); Ain Segoufta, 8 May 1884, *N. Doumet-Adanson et Bonnet s.n.* (P04966853); Gouifla, 9 May 1884, *Doumet-Adanson et Bonnet s.n.* (P04966865); Djebel Tournich, 13 May 1884, *N. Doumet-Adanson et Bonnet s.n.* (P04966863); Bir Beni Zid, 23 May 1884, *N. Doumet-Adanson et Bonnet s.n.* (P04966858); Tunisie (sud), s.d. [XX century], *I. Serres s.n.* (MPU070242); Tunisie, Oued Metlaoui, 19 April 1947, *I. Serres s.n.* (MPU097765); Pont-du-Fahs, 17 June 1950, *L. Faurel s.n.* (P05168533); Tunesien: Sidi bou-Zid, 30 May 1961, *Fr. Zednik s.n.* (W0083399); T. Sebkha au Sud d’El-Djem, s.d. [XIX century], *Pomel s.n.* (MPU097764); Amira-Hatem, Zeramdine, 27 May 2018, *R. El Mokni 6* (Herb. R. El Mokni, SAF); Hajeb El Ayoun, 27 May 2022, *G. Domina*, *R. El Mokni*, *G. Barone s.n.* (Herb. R. El Mokni, SAF); Touza, 24 May 2022, *R. El Mokni s.n.* (Herb. R. El Mokni).

***Dianthus illyricus*** subsp. ***angustifolius*** (Poir.) Fassou, N. Korotkova, Dimop. & Borsch, PhytoKeys 196: 113 (2022)

≡ *Silene angustifolia* Poir., Voy. Barbarie 2: 164. (1789)

≡ *Petrorhagia illyrica* subsp. *angustifolia* (Poir.) P.W.Ball & Heywood in Bull. Brit. Mus. (Nat. Hist.), Bot. 3: 136. 1964.

**Type** (neotype designated by [2] (p. 113)): NORTH AFRICA. *J.L.M. Poiret 32* (P00286897 [digital photo!]).

**Taxonomy and distribution**: Four subspecies of *Dianthus illyricus* are currently accepted [24]: *D. illyricus* subsp. *angustifolius* (occurring in all North African countries); *D. illyricus* subsp. *haynaldianus* (Nyman) Fassou, N. Korotkova, Dimop. & Borsch (occurring in Italy and the Eastern Mediterranean); *D. illyricus* (Ard.) Fassou, N. Korotkova, Dimop. & Borsch subsp. *illyricus* (occurring in the Balkan Peninsula) and *D. illyricus* subsp. *taygeteus* (Boiss.) Fassou, N. Korotkova, Dimop. & Borsch (endemic to Greece and Crete). The subspecies were recently confirmed on a molecular basis by the authors of [2]. They are differentiated from each other by the pubescence of the stem and the size of the calyx [25]. *D. illyricus* subsp. *angustifolius* is distinguished from the other subspecies by its narrower leaves (1–3 mm vs. 3–8 mm).

**Distr. in Tunisia** (Figure 1): Widespread: NE: Bizerta (Mateur); VM: Beja (Dj. Gorra); CB: Nabeul (El Haouaria); TC: Monastir (Mesjed-Aïssa); DT: Le Kef (Table de Jugurtha), Kasserine (Dj. Bireno); K: Jendouba (Dj. Ghorra); TS.

**Ecology and phenology in Tunisian populations:** Sandy and rocky soils and cliffs on carbonate and siliceous substrate within coastal and hilly areas. Flowering from May to July.

**Specimina Visa**: Dj. Ghorra, 26 June 2020, *R. El Mokni s.n.* (Herb. R. El Mokni); Mesjed-Aïssa, 26 May 2022, *G. Domina*, *R. El Mokni*, *G. Barone s.n.* (Herb. R. El Mokni); El Haouaria, 19 July 2023, *R. El Mokni s.n.* (Herb. R. El Mokni); Mateur (Bazina), 23 July 2023, *R. El Mokni s.n.* (Herb. R. El Mokni); Table de Jugurtha, 11 July 2024, *R. El Mokni s.n.* (Herb. R. El Mokni); Dj. Gorra, 27 May 2025, *R. El Mokni s.n.* (Herb. R. El Mokni); Dj. Bireno, 28 May 2025, *R. El Mokni s.n.* (Herb. R. El Mokni); Dyr El Kef, 29 May 2025, *R. El Mokni s.n.* (Herb. R. El Mokni).

***Dianthus nudiflorus*** Griff., Not. Pl. Asiat. 4: 466 (1854)

**Type** (Holotype also reported in [26] (p. 103)): Afghanistan, barren rocky mountains around Otipore, 7 Apr 1839, *W. Griffith 132* (K).

= *Velezia rigida* L., Sp. Pl.: 332 (1753)

Type (lectotype designated by Strid in [27] (p. 1053)): Loefling 307, Herb. Linn. No. 326.1 (LINN).

**Taxonomy and distribution**: The species is traditionally included within *Valezia*. It was included in *Dianthus* following molecular phylogenetic analysis in [2], which demonstrated the monophyly of the two taxa. *D. nudiflorus* is a Euroasiatic species occurring from the Atlantic coast of Europe and North Africa to Central Asia [24].

**Distr. in Tunisia** (Figure 1): Cited as being from Tunisia by [28], without specific locality. The same citation is reported by [11]. We found it in DT: Kasserine & Sidi Bouzid (Dj. Mghila) and Siliana (Dj. Serj).

**Ecology and phenology in Tunisian populations:** Rocky soils in hilly areas. Flowering from April to June.

**Specimina Visa**: Djebel Mghila, 12 April 2012, *R. El Mokni s.n* (Herb. Univ. Carthage); Djebel Serj, 14 April 2012, *R. El Mokni s.n* (Herb. Univ. Carthage).

***Dianthus rupicola*** subsp. ***hermaeensis*** (Coss.) O. Bolòs & Vigo, Bull. Inst. Catalana Hist. Nat. 38: 87 (1974) (Figure 2e)

≡ *D. hermaeensis* Coss., Ill. Fl. Atlant. 121 (1891).

≡ *D. rupicola* var. *hermaeensis* (Coss.) F.N. Williams, J. Linn. Soc. London, 29: 363 (1893).

**Type** (lectotype designated by [29] (p. 26)): TUNISIA. Rochers près du Cap Bon, 17 May 1883, *M. M. E Cosson*, *N. Doumet-Adanson*, *A. Letourneaux*, *V. Reboud*, *G. Barratte*, *E. Bonner s.n.* (P04927153 [digital photo!]).

**Taxonomy and distribution**: *Dianthus rupicola* is a central Mediterranean species [24,30], five subspecies have been distinguished: *D. rupicola* subsp. *aeolicus* (Lojac.) Brullo & Minissale; *D. rupicola* subsp. *bocchorianus* Llorens & Gradaille; *D. rupicola* subsp. *hermaeensis*, *D. rupicola* subsp. *lopadusanus* Brullo & Minissale; *D. rupicola* Biv. subsp. *rupicola*. These subspecies are distinguished geographically, morphologically and from the molecular points of view [29]. *D. rupicola* subsp. *hermaeensis* is strictly endemic to the Cap Bon area.

**Distr. in Tunisia** (Figure 1): CB: Nabeul (El Haouaria, Island of Zembra).

**Ecology and phenology in Tunisian populations**: Carbonate cliffs of the coastal belt. Flowering from May to July.

**Specimina Visa**: Rochers près du Cap Bon, 17/5/1883, *E. Cosson & al. s.n.* (P04927153); Djeziret Djamour [Zembra], 3 July 1884 *N. Doumet-Adanson et E. Bonnet s.n.* (P04927156); Insula Djamour [Zembra], 19 June 1887, *A. Letourneux s.n.* (P04927155); Zembra, 5 June 1888 *E. Cosson et al. s.n.* (P04927152); El Haouaria, 3 June 1949, *M. Couteaux 63T110* (BR0000028287995); Dj. Sidi Abiad, El Haouaria, Cap Bon, 21 April 1963, *G. Pottier Alapetite s.n.* (MPU000759); Zembra island, 37°07′04″ N, 10°47′48″ E, 27 June 2012, *G. Domina s.n.* (PAL108619); Gouv. Nabeul, around top of Cape Bon, 37°04′12″ N, 11°02′33″ E, 26 March 2014 *N. Ardenghi et al. 0305* (MA909015, PAL, W0085600).

**Figure 3:**
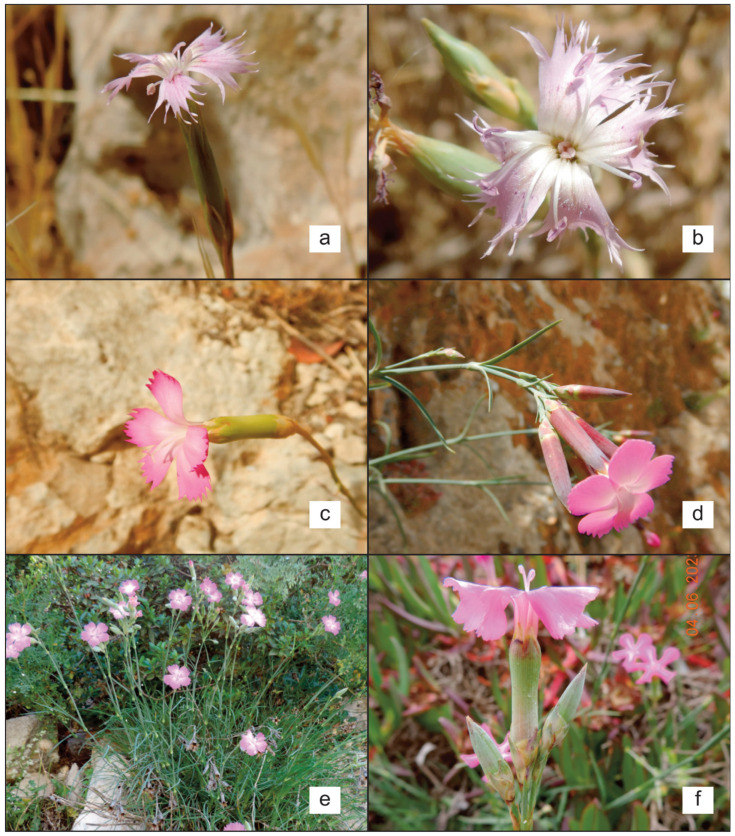
Field photos of *Dianthus* taxa present in Tunisia: (**a**,**b**) *D. serrulatus* in Foum El.Guelta; (**c**) *D. virgineus* var. *virgineus* in Dahmani; (**d**,**e**) *D. virgineus* var. *graminifolius* in Dj. Gorra; (**f**) *D. virgineus* var. *kremeri* in Cap-Blanc (Photos by R. El Mokni).

***Dianthus serrulatus*** Desf., Fl. Atlant. 1: 346 (1798) (Figure 3a,b).

**Ind. Loc.**: [Tunisia] in arenis prope Sfax et Elgem apud Tunetanos.

**Type** (**lectotype designated here**): TUNISIA. R.-L. Desfontaines, Herbier de Barbarie, *Dianthus serrulatus* Desf., Loc: in arenis prope Sfax et Elgem apud Tunetos, Desf. Fl. Atl. I P. 356 tab., Serie de 600 nos donnée par Desfontaines à L.G. Lemonnier; acquise en 1803 par D. Delessert; revue en 1828 et 1829 par Desfontaines, pour servir à illustrer les types décrits dans la Flora Atlantica; intercalée en 1916 dans la collection Générale de l’Herbier Delessert.—Voy. Lasègne: Musée botanique de M. Benjamin Delessert, p. 60. (G00425580 [digital photo!]).

**Nomenclatural notes**: We found one herbarium specimen in G (G00425580) that reported the same locality in the protologue and one specimen in MPU (MPU609808) from the Desfontaines herbarium but without locality and date. We designated the specimen from G as the lectotype of the name. This specimen matches the protologue and corresponds to the current application of the name.

= *D. serrulatus* subsp. *macranthus* Maire, Fl. Afr. Nord 10: 302. 1963.

Ind. Loc.: ALGERIA. Garrouban, Djebel Amour, Djebel Metlili.

**Type** (**lectotype designated here**): ALGERIA. HERBIER POMEL/*Dianthus serrulatus* Desf., Gar-Rouban (Vaissa), 15 September 1899, [*A. Pomels.n.*] (MPU125825 [digital photo!]).

Nomenclatural notes: *Dianthus serrulatus* subsp. *macranthus* was validly published in 1963 with reference to previously published descriptions. In [28] (sub *D. serrulatus* var. *broteroi*) the localities Garrouban, Djebel Amour, Djebel Metlili are given. In [31] (sub *D. fimbriatus* M. Bieb.) a specimen from Ghar-Rouban by Pomel is reported. Here, we designated, as lectotype of the name, a specimen collected in Ghar-Rouban from the herbarium of Pomel.

= *D. serrulatus* var. *subsimplex* F.N. Williams ex Maire Bull. Soc. Hist. Nat. Afrique N. 23: 169. 1932.

Ind. Loc.: Hab. in montibus Zeugitaniae: Djebel Zaghouan (Kralik, Plantae tunetanae, 6 August 1854, in Herb. Cosson).

**Type** (**lectotype designated here**): TUNISIA. Kralik. Pl. Tunetanae, *Dianthus serrulatus* Desf. var., Djebel Zaghouan 6 August 1854, [*L-Kralik s.n.*] (P04927035 [digital photo!], iso: P04927130 [digital photo!]).

Nomenclatural notes: We found in P three specimens collected by Kralik in Zaghouan the 6 August 1854. Here, we selected the specimen P04927035 as the lectotype of the name because it was more complete and in good condition.

= *D. serrulatus* var. *strictus* Maire, Bull. Soc. Hist. Nat. Afrique N. 23: 169. 1932.

Ind. Loc.: “in collibus aridis Tunetiae centralis: inter Suffetulam (Sbeitla) et Foum-el-Guelta! (Letourneux, 15 July 1887)”

Type: not designated.

Nomenclatural notes: This variety was differentiated by [32] from *D. serrulatus* subsp. *serrulatus* s.s. by being glaucous green in colour (not bright green) and by having 2–3 flowers per scape (rather than having many flowers). In the field we observed individuals of different shades of green and with a variable number of flowers.

– *D. serrulatus* subsp. *eu-serrulatus* Maire, Fl. Afrique N. 10: 302 (1963) [nom. illeg.].

Nomenclatural notes: This name was nomenclaturally superfluous when published because it included the type of *D. serrulatus* (see Art. 24.3 [19]).

– *D. serrulatus* subsp. *eu-serrulatus* var. *genuinus* Maire, Fl. Afrique N. 10: 302 (1963) [nom. illeg.].

Nomenclatural notes: This name was nomenclaturally superfluous when published because it included the type of *D. serrulatus* (see Art. 24.3 [19]).

**Taxonomy and distribution**: The two subspecies (*D. serrulatus* subsp. *serrulatus* and *D. serrulatus* subsp. *macranthus*) actually accepted [14,16,23], are not morphologically, geographically or ecologically differentiated to support such a taxonomic rank. The length of the calyx used by [28,32] to distinguish *D. serrulatus* var. *broteroi* (*D. serrulatus* subsp. *macranthus*) from *D. serrulatus* s.s. is not a stable character; we have observed specimens with deeply fringed petals and both long and shorter epicalyxes. The depth of incision of the petal blade is also a variable character within individuals. Species occur from Morocco to Tripolitania [14].

**Distr. in Tunisia** (Figure 1): NE: Bizerta (Dj. Mennzel Roul, Oued Zitoun); DT: Zaghouan (Dj. Zaghouan), Le Kef (Dyr Le Kef); TC: Kairouan (Dj. Toumiet); Kasserine (Fériana, Kasserine, Sbeitla), Siliana (Al Mashraf, Kessera), Sidi Bouzid (Foum El-Guelta à Djebel Meghila, Sidi-Khaled), Sfax; TS: Gafsa (Dj. Cherb).

**Ecology and phenology in Tunisian populations**: Rocky soils, fields and roadsides in coastal, hilly and Mountain belts. Flowering from May to August.

**Specimina Visa:** environs de Sfax, 1856, *Ducouret s.n.* (MPU285196); Djebel Toumiet, 13 May 1884, *N. Doumet-Adanson et E. Bonnet s.n.* (MPU609821); Fériana, 17 June 1884, *J.-F. Robert s.n.* (MPU609820); Fériana, 22 June 1884, *A. Letourneux s.n.* (P04966867); Djebel Zaghouan, 6 August 1844, *L. Kralik s.n.* (P04927130, P04927135); Sfax, 1 June 1854, *L. Kralik s.n.* (P04927139); Sfax, 1 June 1854. *E. Cosson s.n.* (LY0682184); Dj. El Roul, 11 July 1883, *Cosson*, *Doumet*, *Adanson*, *A. Letourneux s.n.* (P04927127, P04927034); Foum El-Guelta (Djebel Meghila), 19 May 1889, *A. Letourneux s.n.* (ANG047105); Sfax, 1 June 1854, *L. Kralik s.n.* (ANG47107, LY0087308, P04927139, P04927044, P05033186); S.O. de Mehedia, 11.6.1883, *E. Cosson*, *N. Doumet-Adanson*, *A. Letourneux*, *V. Rebond*, *G. Barratte*, *E. Bonnet s.n.* (ANG047111, MPU097762, P04966810); Oued Zitoun, 10–11 June 1884, *A. Letourneux s.n.* (P04966862); Djebel Cherb, 12 June 1884, *A. Letourneux s.n.* (ANG047112, MPU610260, P04966808); Djebel Cherb, Oued bufarma, 12 June 1884, *A. Letourneux s.n.* (P04966866); Sfax, 4/1909, *J. A. Battandier s.n.* (MPU097761); Mont Zaghouan, June 1927, *M Ritter s.n.* (MPU610263); Environs de Bossuet, 1300 m, 16 July 1933, *A. Faure s.n.* (IND0088023); Tiaret: vallon de l’Oued-Sidi-Khaled, 1100 m, 27 May 1940, *A. Faure s.n.* (MPU125829); Kessera, 30 October 1949; *Pottier-Alapetite s.n.* (MPU000760); Bou Kornine, 20/11/1949, *Pottier-Alapetite s.n.* (MPU000761); Bou Kornine, 20 November 1949, *Pottier-Alapetite s.n.* (MPU000761); Plateau de Kesra, 19 July 2018, *G. Domina & R. El Mokni s.n.* (SAF); Al Mashraf, 35.932423° N 9.121945° E, 810 m a.s.l., 19 July 2018, *G. Domina & R. El Mokni s.n.* (SAF); Dyr El Kef, 20 July 2018, *G. Domina*, *R. El Mokni s.n.* (SAF); Table de Jugurtha, 11 July 2024, *R. El Mokni s.n.* (Herb. R. El Mokni).

***Dianthus virgineus*** L., Sp. Pl. [Linnaeus] 1: 412 (1753).

–*Dianthus caryophyllus* subsp. *virgineus* sensu auct.

–*Dianthus sylvestris* subsp. *longicaulis* sensu auct.

**Type** (lectotype designated by [9] (p. 1098)): FRANCE. Caryophyllus Syl. repens multi florus Bauh., Monspelii sponte, Burser XI: 99 (UPS No. V-174060).

**Taxonomy and distribution**: Accepting the view of the authors of [33] that the central Mediterranean *Dianthus virgineus* complex is a morphological continuum with inflated higher taxonomy, we nevertheless identified morphological differences that allowed us to distinguish three taxa in Tunisia. To acknowledge these distinctions without contradicting the broader continuum, we propose here the rank of variety for these taxa. *Dianthus virgineus* is currently documented in France, Italy, and Tunisia [33,34]. The examination of material related to Tunisian taxa allows us to confirm its occurrence also in Spain and Algeria. The distribution of *D. virgineus* and its infraspecific variation across the Iberian Peninsula and North Africa requires further critical assessment.

**Distr. in Tunisia** (Figure 1): NE: Ben Arous (Aïn Sabour), Bizerta (Dj. Ichkeul, Dj. Kebir, Cap-Blanc, Sidi Nsir); K: Jendouba (Ghar-Dimaou, Kef el Bled); CB: Nabeul (Korbous); DT: Ben Arous (Dj. Ressas), Gafsa (Dj. Orbata), Le Kef (Dahmani, Djérissa, Dj. Zaafrane, Dyr El Kef, Kalaat Senan, Les Zouarines, Nebeur, Table de Jugurtha, Tajerouine), Kasserine (Dj. Bireno, Dj. Chambi, Dj. Khechem el Kelb), Zaghouan (Dj. Zaghouan), Kasserine & Sidi Bouzid (Dj. Mghila), Siliana (Al Mashraf, Dj. Bargou, Souk el Djemâa), Le Kef (Djérissa, Kalaat Senan); TC: Kasserine (Fériana, Haïdra); TS: Gabès (Matmatas); VM: Beja (Dj. Gorra, Dj. Munchar).

**Ecology and phenology in Tunisian populations**: Cliffs and rocky fields on carbonate and siliceous substrate from the sea level to the mountain belt. Flowering from May to July.

***Dianthus virgineus*** var. ***virgineus*** (Figure 3c)

*= D. siculus* C. Presl in J. & C. Presl, Delic. Prag. 59 (1822).

*= D. sylvestris* subsp. *siculus* (C. Presl) Tutin, Feddes Repert. 68: 190 (1963).

*= D. caryophyllus* subsp. *siculus* (C. Presl) Arcang., Comp. Fl. Ital., ed 2, 306 (1894).

Type (lectotype designated by [35]): ITALY. in pascuis carii Panormi et ad Caltavutturem prope Imeram, Jun–Jul 1817, C. Presl s.n. (PRC).

= *Dianthus godronianus* Jord. in Mém. Acad. Roy. Sci. Lyon, Sect. Sci., ser. 2, 1: 241 (1851).

= *D. caryophyllus* subsp. *virgineus* (L.) Rouy & Fouc. var. *godronianus* (Jord.) Briq., Fl. Corse 1: 574 (1910).

= *D. sylvestris* var. *godronianus* (Jord.) Kerguélen, Lejeunia, nouv. ser., 120: 81 (1987).

= *D. caryophyllus* subsp. *godronianus* (Jord.) P. Martin, Soc. Ech. Pl. Vasc. Eur. Bassin Médit. 19: 93 (1984).

*= D. sylvestris* var. *godronianus* (Jord.) Kerguélen, Lejeunia, Nouv. Sér., 120: 81. 1987.

Type (lectotype designated by [9]): CORSICA. Soleirol, Herb. Cors., 959 *Dianthus virgineus* L. (Gren. et Godr.), *Dianthus sylvestris* Duby, Bastia— May 1823 (P05000349 [digital photo!]).

**Taxonomy and distribution**: *D. virgineus* s.s. occurs in the distribution range of the species. The nomenclatural type [9] includes small-sized individuals, with short leaves and a single flower per floral scape.

**Distr. in Tunisia** (Figure 1): NE: Bizerta (Dj. Kebir); TC: Siliana (Al Mashraf, Souk el Djemâa); DT: Le Kef (Dahmani, Les Zouarines), Kasserine (Dj. Khechem el Kelb), Siliana (Dj. Serj).

**Ecology and phenology in Tunisian populations**: Cliffs and rocky terrain on carbonate and siliceous substrate from the sea level to the mountain belt. Flowering from May to July.

**Specimina Visa**: Camp de Souk-el Djema au nord de Makter, 24 June 1883, *E. Cosson, N. Doumet-Adanson*, *A. Letourneux*, *V. Reboud*, *G. Barratte*, *E. Bonnet s.n.* (P05020444, P05020519); Souk-el Djema, 25 June 1883, *E. Cosson*, *N. Doumet-Adanson*, *A. Letourneux*, *V. Reboud*, *G. Barratte*, *E. Bonnet s.n.* (MPU610271, P05020444); Djebel Khechem el Kelb, 23 June 1884, *A. letourneux s.n.* (P05020465); Djebel Serj, 30 April 2014, *R. El Mokni 2* (Herb. R. El Mokni, SAF); Al Mashraf, 19 July 2018, *G. Domina et R. El Mokni s.n.* (Herb. R. El Mokni, SAF); Dahmani, 20 July 2018, *G. Domina et R. El Mokni s.n.* (Herb. R. El Mokni, SAF); Les Zouarines, 20 July 2018, *G. Domina et R. El Mokni s.n.* (Herb. R. El Mokni, SAF); Djebel Kebir, 22 July 2018, *G. Domina et R. El Mokni s.n.* (Herb. R. El Mokni, SAF).

***Dianthus virgineus*** var. ***graminifolius*** (C.Presl) Domina & El Mokni **comb. nov.** (Figure 3d,e).

≡ *D. graminifolius* C.Presl, Fl. Sicul. [Presl] 1: 147 (1826).

≡ *D. arrostoi* var. *graminifolius* (C.Presl) Lojac., Fl. Sicul. [Lojacono] 1(1): 164 (1889).

**Type** (lectotype designated by [20] (p. 166)): ITALY. In apricis montis Cucii ad Panormum, Jul 1817, Presl (PRC).

*= D. gasparrinii* Guss., Fl. Sicul. Syn. 1: 479 (1843).

= *D. caryophyllus* subsp. *gasparrinii* (Guss.) Arcang., Comp. Fl. Ital., ed. 2: 306. 1894.

Type (lectotype designated by [20](p. 162): ITALY. in collibus aridis argilloso calcarei colline di Polizzi, October, Gasparrini s.n. (NAP).

= *D. boissieri* Willk., Icon. Descr. Pl. Nov. 1: 22 (1853).

*= D. sylvestris* subsp. *boissieri* (Willk.) Dobignard, J. Bot. Soc. Bot. France 20: 37 (2002).

Ind. Loc.: “Habitat *D. Boissieri* in rupestribus calcareis regionis calidae superioris provinciae Malacitanae: circa Alhaurin, Monda et al.ibi, Boissier! Prolongo! ad alt. 900–1000. Floret Junio”

Type (lectotype designated here): SPAIN. H. M. Willkommii iter hispanicum, *Dianthus silvestris* Wulf., hirtus Vill, *boissieri* Wk. n. sp., Hab. in rupestribus regionis calidae superioris prope oppidum Alhaurin in prov. Malacitana, Altitudo: 900, Nom. vulgare: Clavel del Campos, Ex herb. cl. Prolongi, 1845 (COI00059654 [digital photo!]).

Nomenclatural notes: We found in the Willkomm herbarium in COI a specimen collected by P. Prolongo in 1845 (COI00059654), in K two specimens by Boissier dated 1837 (K000725350, K0072531), and in JE a specimen by Boissier without a date (JE00017104) and plate XIII of the *Icones et descriptiones plantarum novarum criticarum et rariorum Europae austro-occidentalis praecipue Hispaniae* [36]. Here, we selected as the lectotype of the name the specimen from the Willkomm herbarium in COI. It is complete in all of its parts, matches the description, and corresponds to the current use of the name.

**Taxonomy and distribution**: This taxon is known from Sicily and Tunisia. It differs from *D. virgineus* var. *virgineus* in that it is a taller plant (40–70 vs. 10–25 cm) with longer leaves (4–12 vs. 1–4.5 cm long) and with flowering scapes with 2–6 flowers (vs. 1–2 flowers in *D. virgineus* var. *virgineus*). Ref. [16] reported a collection of *D. sylvestris* subsp. *boissieri* by L. Dugerdil and S. D. Muller (2024) from Djebel Bargou. The examination of the specimen, kindly made available to the authors, confirmed it as *D. virgineus* var. *graminifolius* and it is indistinguishable from the other Tunisian populations.

**Distr. in Tunisia** (Figure 1): NE: Ben Arous (Aïn Sabour), Bizerta (Dj. Ichkeul, Sidi Nsir); K: Jendouba (Kef el Bled); VM: Beja (Dj. Gorra, Dj. Munchar); CB: Nabeul (Korbous); DT: Ben Arous (Dj. Ressas), Kasserine (Dj. Bireno, Dj. Chambi), Gafsa (Dj. Orbata), Le Kef (Djérissa, Dyr El Kef, Kalaat Senan, Dj. Zaafrane, Nebeur, Table de Jugurtha, Tajerouine), Siliana (Dj. Bargou), Zaghouan (Dj. Zaghouan), Kasserine & Sidi Bouzid (Dj. Mghila); TC: Kasserine (Fériana, Haïdra); TS: Gabes (Matmatas).

**Ecology and phenology in Tunisian populations**: Cliffs and rocky fields on carbonate and siliceous substrate from the sea level to the mountain belt. Flowering from April to July.

**Specimina Visa**: Djebel Zaghouan, 2 July 1854, *L. Kralik 17* (P04966871, P04966911, P05020464); Korba, 22 June 1855, *A. Hénon 73* (P04927120); Zaghouan, 31 May 1883, *E. Cosson*, *N. Doumet-Adanson*, *A. Letourneux*, *G. Barratte*, *E. Bonnet s.n.* (P05020462); Djebel Zaghouan, 1 June 1883, *E. Cosson*, *N. Doumet-Adanson*, *A. Letourneux*, *G. Barratte*, *E. Bonnet s.n.* (P05020455); Djebel Zafran prés Kef, 26 June 1883, *E. Cosson*, *N. Doumet-Adanson*, *A. Letourneux*, *V. Reboud*, *G. Barratte*, *E. Bonnet s.n.* (P05020458); El Kef, 27 June 1883, *E. Cosson*, *N. Doumet-Adanson*, *A. Letourneux*, *V. Reboud*, *G. Battatte*, *E. Bonnet s.n.* (P05020463, P05020520); Guelerat Es Snaum, 29 June 1884, *A. Letourneux s.n.* (P04966157, P04966158, P05020460, P05020521); Haidra, 28 June 1884, *A. Letourneux s.n.* (P05020461); Sidi Gaiez, prope El Kef, 19 May 1886, *A. Letourneux s.n.* (P05020457, P05020466); Djebel Bargou, 2 June 1887, *A. Letourneux s.n.* (P05020446); Djebel Ichkneul, in cacumine, 27 June 1887, *A. Letourneux s.n.* (P04966154); Dj. Recas, 22 May 1888, *G. Barratte s.n.* (P04966134, P04966153); Djebel Ichnel, Hammam El. Ichknel, 15 June 1888, *E. Cosson s.n.* (P05020456); Djebel Ichnel, Hammam El. Ichknel, 19 June 1888, *E. Cosson*, *G. Barratte*, *C. Duval s.n.* (P05020441, P05020442); Dune à l’ouest de l’ embarquement del l’Oued Barka, 21 June 1888, *E. Cosson*, *G. Barratte*, *C. Dival. s.n.* (P05020459); Fériana, 11 April 1912, *S. Buchet s.n.* (P05033181); Djebel Chambi, 19 March 1987, *R. Albert s.n.* (W0087804); Kef, ca. 7 km de Nebeur, 600 m, 21 April 2001, *R. Albert T549* (W0087790); Orbata, 24 April 2017, *R. El Mokni 1* (Herb. R. El Mokni, SAF); Bizerte, 37°19′52″ N 9°51′53″ E, 6 m a.s.l., 22 June 2018, *R. El Mokni 5* (Herb. R. El Mokni, SAF); Jebel Ichkeul, 10 July 2018, *R. El Mokni 4* (Herb. R. El Mokni, SAF); Djebel Zaghouan, 36°22′05″ N 10°7′14″ E, 930 m a.s.l., 18 July 2018, *G. Domina*, *R. El Mokni s.n.* (Herb. R. El Mokni, SAF); Dyr El Kef, 20 July 2018, *G. Domina*, *R. El Mokni s.n.* (Herb. R. El Mokni, SAF); Kef Aïn Sabour, 21 July 2018, *G. Domina*, *R. El Mokni s.n.* (Herb. R. El Mokni, SAF); Al Munshar, 21 July 2018, *G. Domina et R. El Mokni s.n.* (Herb. R. El Mokni, SAF); Sidi Nsir, 21 July 2018, *G. Domina*, *R. El Mokni s.n.* (Herb. R. El Mokni, SAF); Bizerte, 37°19′52″ N, 9°51′53″ E, 6 m a.s.l., 22 July 2018, *G. Domina et R. El Mokni s.n.* (SAF); Fériana, 28 May 2022, *G. Domina*, *R. El Mokni*, *G. Barone s.n.* (SAF); Fériana, 34°57′13″ N, 8°32′51″ E, 820 m a.s.l., 28 May 2022, *G. Domina*, *R. El Mokni*, *G. Barone s.n.* (Herb. R. El Mokni, SAF); Tajerouine, *G. Domina*, *R. El Mokni*, *G. Barone s.n.* (Herb. R. El Mokni, SAF); Al-Jarissah, *G. Domina*, *R. El Mokni*, *G. Barone s.n.* (Herb. R. El Mokni, SAF); Djebel Bargou, 36°41′49″ N 9°36′19″ E, 700 m a.s.l., 29 May 2022, *G. Domina*, *R. El Mokni*, *G. Barone s.n.* (SAF); Table de Jugurtha, 1200 m a.s.l., 29 May 2022, *G. Domina*, *R. El Mokni*, *G. Barone s.n.* (Herb. R. El Mokni, SAF); Ain Gaben, 9 May 2024, *L. Dugerdil et S. D. Muller H3517* (Herb. pers.Dugerdil); Dj. Gorra, 27 May 2025, *R. El Mokni s.n.* (Herb. R. El Mokni), Dj. Bireno, 28 May 2025, *R. El Mokni s.n.* (Herb. R. El Mokni), Dyr Le Kef, 29 May 2025, *R. El Mokni s.n.* (Herb. R. El Mokni).

***Dianthus virgineus*** var. ***kremeri*** (Boiss. & Reut.) Domina & El Mokni **comb. nov.** (Figure 3f)

≡ *D. kremeri* Boiss. & Reut., Pugill. Pl. Afr. Bor. Hispan. 21 (1852).

**Ind. Loc.**: in rupestribus maritimis versus septentrionem spectantibus ad orientem urbis Oran Mauritaniae ubi Jun. floret exemplarque unicum Aprili inuente casu jam floriferum legerunt Boiss. et Reut.

**Type** (**holotype**): ALGERIA. Boissier et Reuter. Iter Algeriensi-Hispanicum, Dianthus, specimen unicum!, Prov. Oran. leger. Reuter, à la grande Falaise, à l’Est d’Oran, April 1849 (G00430757 [digital photo!]).

**Nomenclatural notes**: The indication in the protologue and on the herbarium label, that the specimen is unique, identifies specimen G00430757 as the holotype.

**Figure 4:**
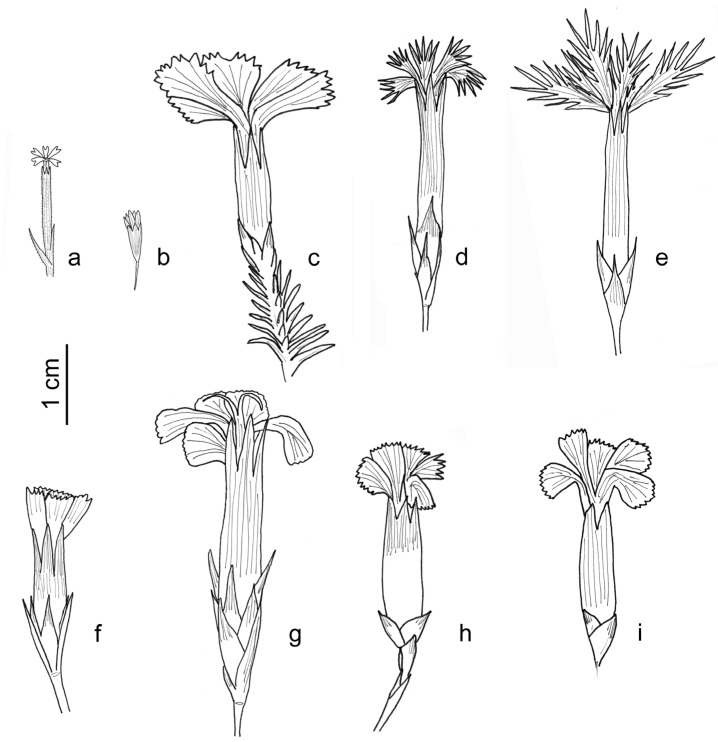
Details of flower structure of *Dianthus* taxa present in Tunisia: (**a**) *D. nudiflorus*; (**b**) *D. illyricus*; (**c**) *D. rupicola* subsp. *hermaeensis*; (**d**) *D. serrulatus*; (**e**) *D. crinitus*; (**f**) *D. cintranus* subsp. *byzacenus*; (**g**) *D. virgineus* var. *kremeri*; (**h**) *D. virgineus* var. *virgineus*; (**i**) *D. virgineus* var. *graminifolius*. Drawings by G. Domina based on original material of the names.

**Taxonomy and distribution**: This taxon occurs in north-western Algeria and more locally in north-eastern Tunisia within rocky calcareous coastal cliffs. The shape and size of the mucro of the epicalyx scales (triangular, 2/3 of the length of the rest of the scale *vs*. linear, 1/5 of the length of the rest of the scale) differentiate *D. virgineus* var. *kremeri* from *D. virgineus* var. *virgineus* and *D. virgineus* var. *graminifolius* (Figure 4).

**Distr. in Tunisia** (Figure 1): NE: Bizerta (Cap-Blanc).

**Ecology and phenology in Tunisian populations**: Cliffs and rocky fields on carbonate and siliceous substrate at the sea level to the mountain belt. Flowering from May to July.

**Specimina Visa**: Bizerta, Cap-Blanc, 22 June 2018, *R. El Mokni s.n.* (Herb. R. El Mokni), *ibidem*, 4 June 2023, *R. El Mokni s.n.* (Herb. R. El Mokni), *ibidem*, 10 May 2025, *R. El Mokni s.n.* (Herb. R. El Mokni).

### 3.2. Taxa Reported in Literature for Tunisia but Not Occurring in the Wild in This Country

***Dianthus balbisii*** Ser. in DC., Prodr. 1: 356 (1824).

**Ind. Loc.**: “Prope Genuam”.

**Type** (**lectotype designated here**): ITALY. Genes, 8 July 1808, *s.c.* [manu *A.P. de Candolle s.n.*] (G00214446 [digital photo!]).

**Nomenclatural notes**: In De Candolle herbarium in G three specimens from Genoa and its surroundings are preserved: Genova (G00214446), Fascia (G00214447) and Portofino (G00214481). Here, we designated the Genova specimen as the lectotype of the name. It is complete in all of its parts and is in full agreement with the protologue.

**Taxonomy and distribution**: We agree with the authors of [2], after analyzing four plastid regions (matK-trnK-psbA, rpl32-trnL, and trnQ-rps16) and nuclear ITS, that this taxon is synonymous with *D. ferrugineus* Mill. Also, the morphological comparison of the type materials of the two taxa did not highlight any difference. *D. balbisii* is endemic to South France and Italy [24]. It was reported from Tunisia [37] without distribution details. This taxon has not been found in the field, nor have specific reports or herbarium specimens been found; therefore, it should be excluded from the flora of Tunisia.

***Dianthus broteroi*** Boiss. & Reut., Pugill. Pl. Afr. Bor. Hispan.: 22. 1852.

*=Dianthus serrulatus* var. *broteroi* (Boiss. & Reut.) Batt. in J.A.Battandier & L.C.Trabut, Fl. Algérie Tunisie: 61. 1902.

Ind. Loc.: Hab. In omni regione calidâ Lusitaniae australis (Brot.) et Hispaniae a Gadbus ad regnum Murcicum (Boiss. Bourg.).

**Type** (**lectotype designated here**): SPAIN. *Dianthus serrulatus* var. *grandiflorus* Boiss. Boissier Voy. Bot. Espagne 1, Table 23 [38].

Nomenclatural notes: In the protologue are cited the specimens by Brotero from southern Portugal, those by Boissier and Bourgeau from Cadiz (SW Spain) and the plate no. 23 of the “Voyage botanique dans le midi de l’Espagne pendant l’année 1837” by [38]. Since we were unable to find any herbarium specimens belonging to the original material, we designate here the plate 23 in [38] as the lectotype of the name. According to Art. 60.8 [19] the spelling of the specific epithet, originally published as “broteri” is to be corrected in “broteroi”. *D. serrulatus* var. *broteroi* was validly published by Battandier by indirect reference to the name of Boissier and Reuter (see Art. 41.3 [19]).

**Taxonomy and distribution**: This taxon is endemic to the southern Iberian Peninsula [39,40]. The taxonomic distinction between *D. broteroi* and *D. serrulatus* was confirmed through molecular studies [40]. Records of *D. broteroi* in North Africa are to be attributed to *D. serrulatus*.

***Dianthus caryophyllus*** L., Sp. Pl. 410. 1753.

**Type** (lectotype designated by [41] (p. 104)): Herb. Linnaeus No. 581.8 (LINN [digital photo!]).

**Taxonomy and distribution**: This species is present in Tunisia only in cultivation. Citations of wild populations for Tunisia have to be referred to *D. virgineus*.

***Dianthus ferrugineus*** Mill., Gard. Dict., ed. 8, n°9 (1768).

**Type** (lectotype designated by [42] (p. 191)): *D. ferrugineus*, s.d., *Miller* (BM000797443 [digital photo!]).

**Taxonomy and distribution**: This taxon is considered endemic to the Iberian Peninsula [24]. See the note on *D. balbisii* for comparison between these two species. Reported with doubt from Tunisa by [12] based on a synonymy with *D. balbisii*. The species was not observed in the field nor in a herbarium. It should be excluded from the flora of Tunisia.

***Dianthus longicaulis*** Ten., Cat. Pl. Hort. Neapol. App. 1, ed. 2, 77 (1819).

≡ *Dianthus sylvestris* subsp. *longicaulis* (Ten.) Greuter & Burdet, Willdenowia 12 (2) 187 (1982).

**Type** (lectotype designated by [20] (p. 158)): ITALY. Camaldoli, Principato Citra, s.d., *Tenore s.n.* (NAP).

**Taxonomy and distribution**: This species is strictly endemic to Southern Apulia [20]. *D. longicaulis* in Tunisia was not observed in the field nor in herbaria. Records of this species for the Tunisian territory should be referred to as *D. virgineus*.

***Dianthus vulturius*** Guss. & Ten., Sem. Hort. Neapol. 3 (1837).

≡ *Dianthus balbisii* subsp. *vulturius* (Guss. & Ten.) Maire, Bull. Soc. Hist. Nat. Afr. Nord 23: 169 (1932).

≡ *Dianthus ferrugineus* subsp. *vulturius* (Guss. & Ten.) Tutin Feddes Repert. Spec. Nov. Regni Veg. 68: 191. 1963.

**Type** (lectotype designated by [43] (p. 308)): ITALY. M. Vulture, ad Pizzo di San Michele, praterie elevate del Vulture, 18 July 1836, *Tenore s.n.* (NAP).

**Taxonomy and distribution**: This species is endemic to southern Italy [44]. It is reported with doubt in [12]; however, it has not been found in the field, nor have specific reports or herbarium specimens been found. Therefore, it is to be excluded from the flora of Tunisia.

**Key to Tunisian taxa of *Dianthus*** (Figure 4, Table 1)
**1.**Epicalyx without scales ***D. nudiflorus*****1.**Epicalyx with 2–8 scales **2****2.**Epicalyx glandular, with five angles, 5–10 mm long***D. illyricus*** subsp. ***angustifolius*****2.**Epicalyx glabrous, cylindrical, more than 15 mm long**3****3.**Perennial sub-shrub plant with woody branches; epicalyx with 10–16 scales***D. rupicola*** subsp. ***hermaeensis*****3.**Annual or perennial plant woody only at the base; epicalyx with 2–8 scales **4****4.**Petals with fringed or lacerated blade for at least 1/3 of their length**5****4.**Petals with entire, crenulated or toothed blade**6****5.**Epicalyx scales terminating gradually into a narrow point (acuminate); petals with limb 7–8 mm long, ciliated up to half its length***D. serrulatus*****5.**Epicalyx scales abruptly terminated by a short point (mucronate); petals with limb 10–12 mm long, ciliated for more than half its length***D. crinitus*****6.**Epicalyx scales terminating gradually into a narrow point (acuminate)***D. cintranus*** subsp. ***byzacenus*****6.**Epicalyx scales abruptly terminated by a short point (mucronate)**7 (*D. virgineus*)****7.**Epicalyx scales terminating gradually into a narrow point (acuminate)***D. virgineus*** var. ***kremeri*****7.**Epicalyx scales abruptly terminated by a short point (mucronate)**8****8.**Plant 10–25 (40) cm tall; leaves 1.0–4.5 cm long; stems 1–2-flowered***D. virgineus*** var. ***virgineus*****8.**Plant 40–70 cm tall; leaves 4–12 cm long; stems 2–5 (6)-flowered***D. virgineus* var. *graminifolius***

## 4. Discussion and Conclusions

This study confirmed the presence of 7 species in Tunisia. The nomenclatural types (lectotypes) of 11 names are designated here. Two subspecies are generally accepted as *Dianthus serrulatus* [14,23,32]. Our field and herbarium studies revealed that the characters used to differentiate these taxa are variable even within populations. Therefore, in our opinion, it is not appropriate to continue adopting this division. For *Dianthus virgineus*, of which three subspecies are recognized in Tunisia [11,14,16,23,32], the rank of variety is proposed. This is because the current division into subspecies is questionable from a geographical point of view (more taxa share the same distribution range) and a morphological one (distinctive characters have overlapping ranges).

The nomenclatural confusions among taxa present in previous flora and checklists were resolved thanks to the designation of nomenclatural types. For *Dianthus,* Tunisia acts as a biogeographical bridge between North Africa, Sicily and Southern Italy. Despite the existence of a targeted network of protected areas in Tunisia [45,46], and the fact that many *Dianthus* populations range within protected areas (e.g., Ichkeul National Park, Zembra National Park, Jebel Mghilla National Park, Jebel Serj National Park, Jebel Zaghouan National Park and Chambi National Park), several grow in vulnerable habitats such as active quarries (e.g., Djérissa, Fériana), agricultural lands (Amira-Hatem, Touza, Zeramdine), and roadsides (e.g Hajeb El Ayoun), where they are threatened due to human activities [47,48]. Most of the investigated populations consist of a few dozen individuals. Some populations occur in restricted military zones or private properties, making monitoring and conservation efforts particularly challenging [49,50]. Regular assessment of population size and trend is crucial to prevent local extinctions [51]; however, accessibility constraints hinder systematic surveys [52]. Urgent conservation measures, including habitat protection and ex situ preservation, are needed for at-risk populations [53,54], especially given the increasing threats from land-use changes and climate variability [55].Further studies should be undertaken for the taxa reported in the Iberian Peninsula, Morocco and Algeria. As previously emphasized by [8,10,33], an integrated approach combining morphology and molecular analysis is valuable for robust taxonomic revisions. To further refine species boundaries and clarify evolutionary relationships within Tunisian *Dianthus* populations, molecular studies—particularly phylogenomic analyses—should augment traditional morphological assessments. Such a combined strategy will not only validate existing taxonomic hypotheses but also uncover potential cryptic diversity and provide deeper insights into the group’s evolutionary history. Future research should prioritize this dual methodology to ensure a comprehensive and accurate understanding of *Dianthus* systematics in Tunisia.

## Figures and Tables

**Table 1:** Synoptic table of *Dianthus* taxa present in Tunisia, their synonyms, biogeographical areas (according to [21]) and habitats.

Taxon	Synonyms	Distribution	Habitats
* Dianthus cintranus subsp. ***byzacenus*** (Burollet) Greuter & Burdet	*D. byzacenus* Burollet; *D. gaditanus* subsp. *byzacenus* (Burollet) Maire; *D. campestris* sensu Barratte in Bonnet & Barratte non M.Bieb.	TC, DT, TS	Rocky soils, fields and roadsides in coastal and hilly areas
***Dianthus crinitus*** Sm.	*D. amoenus* Pomel; *D. crinitus* var. *amoenus* (Pomel) Maire; *D. mesanidum* Litard. & Maire; *D. serratulus* subsp. *macranthus* var. *mesanidum* (Litard. & Maire) Maire; *D. crinitus* var. *flaviflorus* Emb.; *D. crinitus* var. *australis* Maire	TC, DT, TS	Sandy soils, fields and roadsides in coastal and hilly areas
***Dianthus illyricus*** subsp. ***angustifolius*** (Poir.) Fassou, N. Korotkova, Dimop. & Borsch	*Silene angustifolia* Poir.; *Petrorhagia illyrica* subsp. *angustifolia* (Poir.) P.W.Ball & Heywood	K, NE, VM, CB, TC, DT, TS	Rocky soils in coastal and hilly areas.
***Dianthus nudiflorus*** Griff.	*Velezia rigida* L.	DT	Rocky soils in hilly areas
* Dianthus rupicola subsp. ***hermaeensis*** (Coss.) O. Bolòs & Vigo	*D. hermaeensis* Coss.; *D. rupicola* var. *hermaeensis* (Coss.) F.N. Williams	CB	Carbonate cliffs of the coastal belt.
***Dianthus serrulatus*** Desf.	*D. serrulatus* subsp. *macranthus* Maire; *D. serrulatus* var. *subsimplex* F.N. Williams ex Maire; *D. serrulatus* var. *strictus* Maire; *D. serrulatus* subsp. *eu-serrulatus* Maire; *D. serrulatus* subsp. *eu-serrulatus* var. *genuinus* Maire	NE, DT, TS	Rocky soils, fields and roadsides in coastal, hilly and mountain belts.
***Dianthus virgineus*** L. var. ***virgineus***	*D. siculus* C. Presl in J. & C. Presl; *D. sylvestris* subsp. *siculus* (C. Presl) Tutin; *D. caryophyllus* subsp. *siculus* (C. Presl) Arcang.; *D. caryophyllus* subsp. *virgineus* (L.) Rouy & Fouc. var. *godronianus* (Jord.) Briq.; *D. sylvestris* var. *godronianus* (Jord.) Kerguélen; *D. caryophyllus* subsp. *godronianus* (Jord.) P. Martin; *D. sylvestris* var. *godronianus* (Jord.) Kerguélen	NE, TC, DT	Cliffs and rocky fields on carbonate and siliceous substrate from the sea level to the mountain belt
***Dianthus virgineus*** var. ***graminifolius*** (C.Presl) Domina & El Mokni	*D. graminifolius* C.Presl; *D. arrostoi* var. *graminifolius* (C.Presl) Lojac.; *D. gasparrinii* Guss.; *D. caryophyllus* subsp. *gasparrinii* (Guss.) Arcang.; *D. boissieri* Willk.; *D. sylvestris* subsp. *boissieri* (Willk.) Dobignard	K, NE, VM, CB, DT, TC, TS	Cliffs and rocky fields on carbonate and siliceous substrate from the sea level to the mountain belt
***Dianthus virgineus*** var. ***kremeri*** (Boiss. & Reut.) Domina & El Mokni	*D. kremeri* Boiss. & Reut.	NE	Cliffs and rocky fields on carbonate and siliceous substrate at the sea level

* Taxon endemic to Tunisia.

## Data Availability

Data is contained within the article.
